# Supplementary material for: Assessment of antenatal care quality in Ethiopia: Facility-based study using service provision assessment data
Source: PLoS One. 2025 Jan 16;20(1):e0313527. doi: 10.1371/journal.pone.0313527 (PMC11737742; doi:10.1371/journal.pone.0313527)
Supplement: S1 Table — (DOCX) [file pone.0313527.s001.docx]

**S1 table: Descriptions of the independent variables, ESPA 2021- 2022**

| **Women-related variable** | **Value levels** | **Descriptions** |
| --- | --- | --- |
| Maternal age | Continuous | Maternal age in completed years |
| Gestational age | Continuous | Gestational age in weeks calculated by providers |
| Gravidity | 1. Primigravida 2. Multigravida |  |
| Marital status | 1. Unmarried 2. Married | Women who are divorced/separated/widowed are considered unmarried |
| Educational status | 0. Never attended school  1. Primary  2. Secondary  3. College & above |  |
| Area of residence | 1.Urban  2. Rural |  |
| Partner involvement during ANC | 0.No  1. Yes | Partners who involved or had history of involvement during ANC were considered as involved. |
| Covered by healthcare insurance | 1.Yes  2.No |  |
| **Healthcare provider and facility variable** | **Value levels** | **Descriptions** |
| Region | 1.Addis Ababa  2.Amhara  3.Oromia  4.Somali  5.SNNP  6.Sidama  7.Other | Afar, Benshangul Gumuz and Harari, Dire Dawa and Gambella were considered as other |
| Qualification of the care provider | 1. Medical doctors 2. Health officer 3. Nurse 4. Midwife 5. Laboratory professional 6. Other | Other includes integrated emergency surgical officer, health extension worker level 3 &4 and other clinical staffs |
| Healthcare facility nearest to home | 1.Yes  2. No |  |
| Healthcare facility location | 1.Urban |  |
|  | 2.Rural |  |
| Care provider’s gender | 1.Male  2.Female |  |
| Type of healthcare facility | 1.Hospital  2.Health centre  3.Clinic  4.Health post | Hospital includes referral, general and primary hospital. Clinic includes higher, medium, lower and speciality clinic |
| Facility ownership | 1.Public  2.Other | Other includes Other governmental (military, prison, private for profit) and ngo (mission/faith-based, non-profit) |
