# Supplementary material for: Assessment of antenatal care quality in Ethiopia: Facility-based study using service provision assessment data
Source: PLoS One. 2025 Jan 16;20(1):e0313527. doi: 10.1371/journal.pone.0313527 (PMC11737742; doi:10.1371/journal.pone.0313527)
Supplement: Attachment — Submitted filename: reviewers comments.pdf [file pone.0313527.s002.pdf]

Dear Editors and Authors,

Thank you for giving me the opportunity to review the manuscript, titled “Assessment of antenatal care quality in Ethiopia: Facility based study using service provision assessment data”

The authors of the study chose a very important topic related to maternal and child health.

The study aimed to assess the quality of ANC and its associated factors in Ethiopia. The authors used data the 2021/2022 Ethiopian Service Provision Assessment survey and used data from pregnant women during their first ANC consultation. The authors assessed and summed 24 components of ANC components (ranged 0-24), with higher score indicating a better ANC quality. They employed and fitted a poisson regression model to examine predictors of ANC quality.

The authors found that the mean ANC quality was 11 (SD 3.8), with blood pressure measurement the most commonly performed ANC service, and breast examination for cancer screening the least performed component of ANC. They found that when ANC was provided by female health workers the ANC quality was higher, and that the ANC quality was lower in rural health facilities, and for women with increased gestational age, and for women who received care from non-nearby health facility.

My assessment of the manuscript is that it is well written and is well organized. The findings from this study have the potential to impact health interventions and policy to improve neonatal, children, and women’s health. However, the authors need to address the following two issues before the paper can be considered for publication.

1. In table 3, the column showing regression coefficients (SEs) is not necessary. The authors may opt to revise table 3 which should present IRR (95%CI) and p-values for unadjusted IRRs, and adjusted IRRs (which are the current IRRs in table 3).
2. The flow chart shows that out of 4355 women, data from 2042 women were analysed and 2313 women were excluded. In the discussion section as part of the limitations of the study, the authors may opt to discuss the extent of the possible selection bias because of the exclusion of 2313 women. It would be useful to provide some descriptive statistics similar to the one in table 1, as supplementary material, on the 2313 women who were excluded from this study.
