# Supplementary material for: Assessment of antenatal care quality in Ethiopia: Facility-based study using service provision assessment data
Source: PLoS One. 2025 Jan 16;20(1):e0313527. doi: 10.1371/journal.pone.0313527 (PMC11737742; doi:10.1371/journal.pone.0313527)
Supplement: Attachment — Submitted filename: Comment to the authorPLOS ONE1.pdf [file pone.0313527.s003.pdf]

Peer Evaluation for the Authors:	Original Submission<br>**Assessment of antenatal care quality in Ethiopia: Facility based study using service provision assessment data**<br><br>**Addisu Alehegn Alemu1,2*, Alec Welsh3,4, Theodros Getachew5 , Marjan Khajehei2,6,7,8**
First and foremost, thank you for the opportunity to review your manuscript. This study has significant potential to impact antenatal care quality improvement on a large scale in Ethiopia. I trust that the review process will contribute to further enhancing the manuscript and increasing its likelihood of reader understanding in this context.<br><br>The paper is well-constructed and presents a comprehensive study with several notable strengths, including a well-crafted title and clear organization. However, it would benefit from the inclusion of a conceptual framework to more effectively link the variables. Additionally, the claims should be more clearly positioned within the context of existing literature, supported by relevant citations. The feedback provided aims to assist in refining your work and enhancing its impact within the field.	
Abstract:	Comments
Background:<br><br>More detail in the similar article in<br><br>**https://**doi: 10.1177/17455065221076731<br><br>https://doi.org/10.1155/2020/5436324<br><br>https://doi: 10.7189/jogh.07.021101.	The background section requires more detail regarding the Ethiopian Service Provision Assessment (ESPA) for 2021-2022 and the WHO ANC guidelines from 2016, along with their updates. The authors should clearly identify the main problems in Ethiopia, particularly maternal and neonatal morbidity and mortality rates linked to communicable diseases and undernutrition over the past 3-5 years.<br><br>It is essential to highlight the specific challenges or gaps in health services, including health education, screening, disease prevention, and respondent characteristics like age, residence, education, wealth, and ANC visits. The SPA should also address issues within the healthcare delivery system,(that it is not seen on the study) such as: Facility Infrastructure: Staffing and Training: Availability of Equipment and Supplies: Service Delivery Processes: Adherence to Clinical Guidelines: and Health Information Systems: Accuracy in patient records, data tracking, and its use in improving maternal care quality.<br><br>The authors should refine the conceptual framework of the ESPA to more effectively assess ANC quality as informed by DHS survey data.
Methods	The methods section indicates the use of DHS data. The authors should provide a clear justification for the selection of survey components and factors analyzed in the study. Additionally, they should ensure that these choices are consistently aligned with related literature to better support the study's objectives and enhance the overall clarity and coherence of the research methodology.

Results	The authors have effectively presented their findings across key areas, including the characteristics of pregnant women (Table 1), the quality of antenatal care (ANC) (Table 2), and the determinants of ANC quality (Table 3). The results comprehensively address critical factors such as rural versus urban residency, regional variations, the proximity of healthcare facilities to patients' homes, the gender of the care providers, and gestational age.<br><br>These factors are crucial in understanding the disparities and determinants of ANC quality in Ethiopia. The inclusion of these variables provides a robust framework for interpreting the quality of care provided to pregnant women and highlights areas that require targeted interventions. The results clearly indicate how these factors are associated with the quality of ANC, thereby offering valuable insights for policymakers and healthcare providers aiming to improve maternal health outcomes.
Discussion	The authors have provided a detailed discussion in the areas of pregnant women's characteristics (Table 1), the quality of antenatal care (ANC) (Table 2), and the determinants of ANC quality (Table 3). However, the discussion could be more coherent by better linking these findings throughout the manuscript.<br><br>In particular, the factors such as rural residency, region, proximity of the healthcare facility to the home, care provider's gender, and gestational age should be more clearly connected in the discussion to demonstrate their impact on ANC quality in Ethiopia. The manuscript should emphasize how these factors significantly influence the quality of ANC, as evidenced by the present study, and how these findings compare or contrast with previous studies.<br><br>For instance, the significant factors identified, including gestational age, region, distance from the healthcare facility, care provider's gender, type of facility, and location, as highlighted in Table 2, should be discussed in a way that clarifies their role in determining ANC quality. Additionally, it would be beneficial to explain how these factors are not only associated with the quality of ANC but also how they might not serve as determinants in other contexts or studies.<br><br>By strengthening these linkages and ensuring that the discussion clearly aligns with the study's findings, the manuscript will provide a more robust and comprehensible narrative on the quality of ANC in Ethiopia.

Strangles and Limitations	The strengths of this study are well-articulated, particularly in the use of the most recent nationally representative healthcare facility survey data and the rigorous methodology, including the employment of standardized tools and trained data collectors. The use of a standardized checklist recognized as the gold standard for evaluating actual service provision adds robustness to the study's findings.<br><br>However, the limitations also warrant careful consideration. The potential for the Hawthorne effect, where healthcare providers alter their behavior due to awareness of being observed, could indeed influence the study's results, possibly leading to an overestimation of the quality of care. The exclusion of certain populations, such as those from the Tigray region and women not attending their first ANC visit, limits the generalizability of the findings across all contexts in Ethiopia. Additionally, the reliance on secondary data inherently restricts the study to the available ANC components, and the study's observational nature limits its ability to establish causation. Future research could address these limitations by incorporating a broader sample and employing methodologies that allow for causal inference.
Conclusion	The conclusion effectively aligns with the study's objectives, needs to be concise, clearly summarizing the key findings and providing actionable recommendations. The identification of regional disparities and the influence of healthcare provider gender on ANC quality is particularly valuable. The recommendation to prioritize resource allocation in rural and under-resourced facilities is both practical and necessary, given the study's findings.<br><br>However, to enhance the impact of the study, the conclusion could be further refined to emphasize the urgency of addressing these disparities in ANC quality, especially in the context of Ethiopia's high maternal and child morbidity and mortality rates. Additionally, while the recommendation for qualitative research on the gender of healthcare providers is appropriate, it would be beneficial to briefly suggest specific areas or questions this research should focus on, to guide future studies.
