# Supplementary material for: Assessment of antenatal care quality in Ethiopia: Facility-based study using service provision assessment data
Source: PLoS One. 2025 Jan 16;20(1):e0313527. doi: 10.1371/journal.pone.0313527 (PMC11737742; doi:10.1371/journal.pone.0313527)
Supplement: Attachment — Submitted filename: Reviewer Recommendation .pdf [file pone.0313527.s005.pdf]

**Reviewer Recommendation and Comments for Manuscript Number PONE-D-24-30395R1: Assessment of antenatal care quality in Ethiopia: Facility based study using service provision assessment data: Plos ONE**

---

Thank you to the authors and editors for the opportunity to review the manuscript titled, "Assessment of antenatal care quality in Ethiopia: Facility-based study using service provision assessment data." This is an important study on a critical topic related to maternal and child health, especially considering its large-scale scope.

Most of the previous comments have been addressed, and the manuscript has significantly improved. However, there are a few areas that still require refinement:

Abstract:
In the conclusion, the phrase "Thus, efforts are needed to ensure that quality ANC is provided for every pregnant woman in Ethiopia" could be further improved by providing more specific guidance for policymakers and authorities. It would be helpful to include concise recommendations based on the study's findings, focusing on how to improve ANC quality in the Ethiopian context in line with the study's aim of assessing healthcare facility capacity.

Introduction:
This section has improved compared to the previous version. However, the authors should refine the conceptual framework of the ESPA to better align with ANC quality assessment as informed by DHS survey data. This would help readers better understand the scope of the study.

Methods:
The constructs in this section have been improved significantly.

Results:
No additional comments.

Discussion:
The discussion needs to better connect and justify the study's findings. For instance, in line 272, the statement "suboptimal facility readiness, primarily caused by shortages of trained staff and equipment" should be revisited to ensure clarity and better integration with the overall discussion. Additionally, the subsequent sentences regarding WHO recommendations (lines 273-277) do not clearly link to the prior argument. These points should be reworded or modified to create a smoother flow and ensure coherence in presenting the study's implications.

In lines 278-287, the discussion on the frequency and quality of the first ANC visit needs further refinement. The authors should better articulate why and how to increase the

number of pregnant women receiving their first ANC check-up before three months of gestation, using insights from the observed results in Table 1 (Characteristics of the study participants).

For example, factors such as "Partner’s involvement" could be highlighted as a potential strategy to encourage earlier ANC visits. Additionally, Table 2 presents critical data, such as:

Discussed healthy pregnancy: 39.0%
Discussed healthy diet: 47.4%
Discussed importance of at least 4 ANC visits: 19.0%
The relatively low percentages across these indicators underscore the need for improved ANC quality. The discussion should address these gaps and suggest specific interventions, such as better communication during consultations or enhanced health education, to promote early and frequent ANC attendance.

Strengthening this section will improve the manuscript’s clarity on how to address the barriers to early ANC and ensure alignment with the study's findings.

In line 307, the authors mention that "there is evidence that availability of resources does not guarantee provision of quality ANC [59]." However, this statement seems disconnected from the previous assertion in line 272, where it is suggested that "The low ANC content observed in our study could be due to suboptimal facility readiness, primarily caused by shortages of trained staff and equipment [45]."

To improve coherence, the authors should clarify the relationship between resource availability and ANC quality. If resource availability alone is not sufficient for ensuring quality care, the conclusion should focus more on other factors—such as the provider's gender or the proper use of resources—that contribute to the low quality of ANC. The authors should also provide clearer evidence or examples from their findings to support this argument, ensuring alignment between both statements. This refinement will strengthen the discussion and offer a more nuanced understanding of the factors influencing ANC quality.

Additionally, the conclusion seem so long , it should be more concise and clear.
