# Supplementary material for: Assessment of antenatal care quality in Ethiopia: Facility-based study using service provision assessment data
Source: PLoS One. 2025 Jan 16;20(1):e0313527. doi: 10.1371/journal.pone.0313527 (PMC11737742; doi:10.1371/journal.pone.0313527)
Supplement: Attachment — Submitted filename: Point by point response.docx [file pone.0313527.s004.docx]

***Dear Editor and Reviewers,***

First, we would like to express our gratitude to the journal editor and the reviewers for their constructive feedback. We have addressed all the concerns raised by the editor and reviewers and have thoroughly revised the manuscript. We believe it is now in a much-improved state. Below, you will find our detailed, point-by-point response. If you have any further concerns, please feel free to contact the corresponding author.

**Response to the Editor**

When submitting your revision, we need you to address these additional requirements.

1. Please ensure that your manuscript meets PLOS ONE's style requirements, including those for file naming. The PLOS ONE style templates can be found at 
<https://journals.plos.org/plosone/s/file?id=wjVg/PLOSOne_formatting_sample_main_body.pdf> and 
<https://journals.plos.org/plosone/s/file?id=ba62/PLOSOne_formatting_sample_title_authors_affiliations.pdf>

2. Your ethics statement should only appear in the Methods section of your manuscript. If your ethics statement is written in any section besides the Methods, please move it to the Methods section and delete it from any other section. Please ensure that your ethics statement is included in your manuscript, as the ethics statement entered into the online submission form will not be published alongside your manuscript.

3. Please include captions for your Supporting Information files at the end of your manuscript, and update any in-text citations to match accordingly. Please see our Supporting Information guidelines for more information: <http://journals.plos.org/plosone/s/supporting-information>. 

Additional Editor Comments:

Overall, this manuscript presents an important topic and employs appropriate methods for its analysis. However, major revisions are required before being acceptable for publication. In your revision, please consider my suggestions as well as the suggestions provided by Reviewer s for further consideration.
1- Please proofread the whole article for language correction (Last line in abstract does not have a punctuation).

2- It has been suggested that 99% of maternal deaths in LMICs, including Sub-Saharan Africa, can be prevented with quality ANC [10, 11]. Please check your citations. I could not find this claim it the mentioned citations. Please use appropriate citations or remove this sentence.
3- Discussion: Recommendations for each of the key observations they have made.
4- I propose the authors use, consult, and add the following reference in the manuscript that assess ANC quality in another LMIC.
Stanikzai, M.H., Tawfiq, E., Jafari, M. et al. Contents of antenatal care services in Afghanistan: findings from the national health survey 2018. BMC Public Health 23, 2469(2023). <https://doi.org/10.1186/s12889-023-17411-y>
5- Please revise your tables according the the journal style.
6- Please add line numbers in the revised manuscript.

**Response:** Thank you very much for the valuable suggestions and the recommendation to consult the manuscript. We have made significant revisions to the background section, taking into account the editor’s and one of the reviewer’s suggestions. We have also aligned our study with the journal’s guidelines. Additionally, we received support from SuperScript Writing and Editing.

**Response to the Reviewers**

**Reviewer-1**

1. In table 3, the column showing regression coefficients (SEs) is not necessary. The authors may opt to revise table 3 which should present IRR (95%CI) and p-values for unadjusted IRRs, and adjusted IRRs (which are the current IRRs in table 3).

**Response:** The comment has been accepted, and we have thoroughly revised the table to incorporate the necessary changes.

2. The flow chart shows that out of 4355 women, data from 2042 women were analysed and 2313 women were excluded. In the discussion section as part of the limitations of the study, the authors may opt to discuss the extent of the possible selection bias because of the exclusion of 2313 women. It would be useful to provide some descriptive statistics similar to the one in table 1, as supplementary material, on the 2313 women who were excluded from this study.

**Response:** We acknowledge the reviewer’s suggestion to clearly state the excluded population. We would like to assure the reviewer that we used secondary data collected from randomly selected pregnant women across the country. We utilized data exclusively from participants who were included in the study and had received their first ANC visit. This is because the interventions and procedures recommended for pregnant women vary based on the number of ANC visits, with the first visit covering a broader and more comprehensive range of services compared to subsequent visits. While this approach minimizes selection bias, it may limit the generalizability of our study's findings regarding the quality of service across the country. Considering the reviewer’s concern, we have addressed our reasons for selecting the participants in the sampling section and discussed the limitations in the limitations section.

**Reviewer-2**

1. **Background**: The background section requires more detail regarding the Ethiopian Service Provision Assessment (ESPA) for 2021-2022 and the WHO ANC guidelines from 2016, along with their updates. The authors should clearly identify the main problems in Ethiopia, particularly maternal and neonatal morbidity and mortality rates linked to communicable diseases and undernutrition over the past 3-5 years. It is essential to highlight the specific challenges or gaps in health services, including health education, screening, disease prevention, and respondent characteristics like age, residence, education, wealth, and ANC visits. The SPA should also address issues within the healthcare delivery system, (that it is not seen on the study) such as: Facility Infrastructure: Staffing and Training: Availability of Equipment and Supplies: Service Delivery Processes: Adherence to Clinical Guidelines: and Health Information Systems: Accuracy in patient records, data tracking, and its use in improving maternal care quality. The authors should refine the conceptual framework of the ESPA to more effectively assess ANC quality as informed by DHS survey data.

**Response:** Thank you for your insightful suggestions to enhance the background section. We have made substantial revisions based on your input and believe you will find the updated section greatly improved.

**Methods:** The methods section indicates the use of DHS data. The authors should provide a clear justification for the selection of survey components and factors analyzed in the study. Additionally, they should ensure that these choices are consistently aligned with related literature to better support the study's objectives and enhance the overall clarity and coherence of the research methodology.

**Response:** Thank you very much for your feedback on clearly stating the methodology we employed. We have made improvements based on your suggestions and have also moved some information from the methods section to the background section.

2. **Results**: The authors have effectively presented their findings across key areas, including the characteristics of pregnant women (Table 1), the quality of antenatal care (ANC) (Table 2), and the determinants of ANC quality (Table 3). The results comprehensively address critical factors such as rural versus urban residency, regional variations, the proximity of healthcare facilities to patients' homes, the gender of the care providers, and gestational age. These factors are crucial in understanding the disparities and determinants of ANC quality in Ethiopia. The inclusion of these variables provides a robust framework for interpreting the quality of care provided to pregnant women and highlights areas that require targeted interventions. The results clearly indicate how these factors are associated with the quality of ANC, thereby offering valuable
   insights for policymakers and healthcare providers aiming to improve
   maternal health outcomes

**Response:** Thank you very much for your scholarly

3. **Discussion:** The authors have provided a detailed discussion in the areas of
   pregnant women's characteristics (Table 1), the quality of antenatal care (ANC) (Table 2), and the determinants of ANC quality (Table 3). However, the discussion could be more coherent by better linking these findings throughout the manuscript. In particular, the factors such as rural residency, region, proximity of the healthcare facility to the home, care provider’s gender, and gestational age should be more clearly connected in the discussion to demonstrate their impact on ANC quality in Ethiopia. The manuscript should emphasize how these factors significantly influence the quality of ANC, as evidenced by the present study, and how these findings compare or contrast with previous studies. For instance, the significant factors identified, including gestational age, region, distance from the healthcare facility, care provider’s gender, type of facility, and location, as highlighted in Table 2, should be discussed in a way that clarifies their role in determining ANC quality. Additionally, it would be beneficial to explain how these factors are not only associated with the quality of ANC but also how they might not serve as determinants in other contexts or studies. By strengthening these linkages and ensuring that the discussion clearly aligns with the study's findings, the manuscript will provide a more robust and comprehensible narrative on the quality of ANC in
   Ethiopia.

**Response:** We appreciate the reviewer’s valuable feedback. We have made efforts to improve the discussion section by incorporating his/her suggestions while preserving the original content. We believe you will find it much improved

**Strength and Limitations**: The strengths of this study are well-articulated, particularly in the use of the most recent nationally representative healthcare facility survey data and the rigorous methodology, including the employment of standardized tools and trained data collectors. The use of a standardized checklist recognized as the gold standard for evaluating actual service provision adds robustness to the study’s findings. However, the limitations also warrant careful consideration. The potential for the Hawthorne effect, where healthcare providers alter their behaviour due to awareness of being observed, could indeed influence the study's results, possibly leading to an overestimation of the quality of care. The exclusion of certain populations, such as those from the Tigray region and women not attending their first ANC visit, limits the generalizability of the findings across all contexts in Ethiopia. Additionally, the reliance on secondary data inherently restricts the study to the available ANC components, and the study’s observational nature limits its ability to establish causation. Future research could address these limitations by incorporating a broader sample and employing methodologies that allow for causal inference.

**Response:** We have incorporated all the suggestions to address the limitations of the study

**Conclusion:** The conclusion effectively aligns with the study’s objectives, needs to
be concise, clearly summarizing the key findings and providing actionable recommendations. The identification of regional disparities and the influence of healthcare provider gender on ANC quality is particularly valuable. The recommendation to prioritize resource allocation in rural and under-resourced facilities is both practical and necessary, given the study’s findings. However, to enhance the impact of the study, the conclusion could be further refined to emphasize the urgency of addressing these disparities in ANC quality, especially in the context of Ethiopia’s high maternal and child morbidity and mortality rates. Additionally, while the recommendation for qualitative research on the gender of healthcare providers is appropriate, it would be beneficial to briefly suggest specific areas or questions this research should focus on, to guide future studies.

**Response:** We appreciate the reviewer’s valuable suggestion to present our study’s conclusion more precisely. We have made it more concise and incorporated the suggested changes.
