# Supplementary material for: Assessment of antenatal care quality in Ethiopia: Facility-based study using service provision assessment data
Source: PLoS One. 2025 Jan 16;20(1):e0313527. doi: 10.1371/journal.pone.0313527 (PMC11737742; doi:10.1371/journal.pone.0313527)
Supplement: Attachment — Submitted filename: Point by point response.docx [file pone.0313527.s006.docx]

***Dear Editor and Reviewers,***

First, we would like to express our gratitude to the journal editor and the reviewer for their feedback in improving our paper. We have addressed all the concerns, and we believe it is now in a much-improved state. Below, you will find our detailed point-by-point response. If you have any further concerns, please feel free to contact the corresponding author

**Response to the Reviewer’s**

1. **Abstract:** In the conclusion, the phrase “Thus, efforts are needed to ensure that quality ANC is provided for every pregnant woman in Ethiopia” could be further improved by providing more specific guidance for policymakers and authorities. It would be helpful to include concise recommendations based on the study's findings, focusing on how to improve ANC quality in the Ethiopian context in line with the study’s aim of assessing healthcare facility capacity

**Response:** Thank you very much for your insightful suggestion. We have revised the conclusion to emphasize the recommendations; however, we removed the associated factors to meet the journal's word limit.

2. **Introduction:** This section has improved compared to the previous version. However, the authors should refine the conceptual framework of the ESPA to better align with ANC quality assessment as informed by DHS survey data. This would help readers better understand the scope of the study

**Response:** We greatly appreciate your suggestion to describe the conceptual framework we employed, and we have now included that description.

3. **Discussion:** The discussion needs to better connect and justify the study’s findings. For instance, in line 272, the statement “suboptimal facility readiness, primarily caused by shortages of trained staff and equipment” should be revisited to ensure clarity and better integration with the overall discussion. Additionally, the subsequent sentences regarding WHO recommendations (lines 273-277) do not clearly link to the prior argument. These points should be reworded or modified to create a smoother flow and ensure coherence in presenting the study's implications. . Additionally, Table 2 presents critical data, such as:

Discussed healthy pregnancy: 39.0%

Discussed healthy diet: 47.4%

Discussed importance of at least 4 ANC visits: 19.0%

The relatively low percentages across these indicators underscore the need for improved ANC quality. The discussion should address these gaps and suggest specific interventions, such as better communication during consultations or enhanced health education, to promote early and frequent ANC attendance

**Response:** We also made major revisions to this paragraph to clarify it and discuss the most important ANC components that were not provided to the majority of pregnant women.

In lines 278-287, the discussion on the frequency and quality of the first ANC visit needs further refinement. The authors should better articulate why and how to increase the number of pregnant women receiving their first ANC check-up before three months of gestation, using insights from the observed results in Table 1 (Characteristics of the study participants).

For example, factors such as "Partner’s involvement" could be highlighted as a potential strategy to encourage earlier ANC visits

**Response:** We suggested strategies to increase early ANC initiation, as recommended by other studies

In line 307, the authors mention that "there is evidence that availability of resources does not guarantee provision of quality ANC [59]." However, this statement seems disconnected from the previous assertion in line 272, where it is suggested that "The low ANC content observed in our study could be due to suboptimal facility readiness, primarily caused by shortages of trained staff and equipment [45]."

To improve coherence, the authors should clarify the relationship between resource
availability and ANC quality. If resource availability alone is not sufficient for ensuring
quality care, the conclusion should focus more on other factors—such as the provider's gender or the proper use of resources—that contribute to the low quality of ANC. The authors should also provide clearer evidence or examples from their findings to support this argument, ensuring alignment between both statements. This refinement will strengthen the discussion and offer a more nuanced understanding of the factors influencing ANC quality

Additionally, the conclusion seems so long, it should be more concise and clearer.

**Response:** Thank you very much for your suggestion to make the discussion more coherent. To do this, we preferred your first recommendation to revise the conclusion to state, ‘’Ensuring both the availability and proper utilization of ANC resources is recommended’’. We did this because the evidence shows that the availability of resources does not guarantee the provision of quality ANC.

4. Additionally, the conclusion seem so long , it should be more concise and clear.

**Response:** we amended it.
